# Hydrogen-Rich Water Improves Cognitive Ability and Induces Antioxidative, Antiapoptotic, and Anti-Inflammatory Effects in an Acute Ischemia-Reperfusion Injury Mouse Model

**DOI:** 10.1155/2021/9956938

**Published:** 2021-10-27

**Authors:** Dain Lee, Jong-Il Choi

**Affiliations:** ^1^Department of Neurosurgery, Hallym University Gangnam Sacred Heart Hospital, Seoul, Republic of Korea; ^2^KU-KIST Graduate School of Converging Science and Technology, Korea University, Seoul, Republic of Korea; ^3^Department of Neurosurgery, Korea University Ansan Hospital, Ansan, Republic of Korea

## Abstract

**Background:**

Cerebral ischemia and its reperfusion injury facilitate serious neurodegenerative diseases such as dementia due to cell death; however, there is currently no treatment for it. Reactive oxygen species is one of the many factors that induce and worsen the development of such diseases, and it can be targeted by hydrogen treatment. This study examined the effect of molecular hydrogen in cerebral ischemia-reperfusion injury, which is emerging as a novel therapeutic agent for various diseases.

**Methods:**

Ischemia-reperfusion injury was generated through bilateral common carotid artery occlusion in C57BL/6 mice. The test group received hydrogen-rich water orally during the test period. To confirm model establishment and the effect of hydrogen treatment, behavioural tests, biochemical assays, immunofluorescence microscopy, and cytokine assays were conducted.

**Results:**

Open field and novel object recognition tests revealed that the hydrogen-treated group had improved cognitive function and anxiety levels compared to the nontreated group, while hematoxylin and eosin stain showed abundant pyknotic cells in a model mouse brain, and this was attenuated in the hydrogen-treated mouse brain. Total antioxidant capacity and thiobarbituric acid reactive substance assays revealed that hydrogen treatment induced antioxidative effects in the mouse brain. Immunofluorescence microscopy revealed attenuated apoptosis in the striatum, cerebral cortex, and hippocampus of hydrogen-treated mice. Western blotting showed that hydrogen treatment reduced Bax and TNF*α* levels. Finally, cytokine assays showed that IL-2 and IL-10 levels significantly differed between the hydrogen-treated and nontreated groups.

**Conclusion:**

Hydrogen treatment could potentially be a future therapeutic strategy for ischemia and its derived neurodegenerative diseases by improving cognitive abilities and inducing antioxidative and antiapoptotic effects. Hydrogen treatment also decreased Bax and TNF*α* levels and induced an anti-inflammatory response via regulation of IL-2 and IL-10. These results will serve as a milestone for future studies intended to reveal the mechanism of action of molecular hydrogen in neurodegenerative diseases.

## 1. Introduction

Cerebral ischemia is one of the fatal diseases that frequently occurs among elderly people and causes diverse disabilities in patients around the world [1–3]. Cerebral ischemia develops due to dysfunctional blood flow, induced either by a blood clot with infarcts or hemorrhages from the damaged blood artery, and it is often accompanied with diabetes or aging [4, 5]. Cerebral ischemia can trigger other neurological diseases such as stroke or dementia, depending on the site of impairment [6–8]. Ischemic stroke is the most prevalent subtype of stroke, which is the fifth leading cause of death among elderly people in the United States; additionally, it is the second leading cause of acquired physical disability in adults [9–11]. Ischemia also increases the risk factors of vascular dementia by hippocampal brain damage in which pyramidal neurons in the CA1 area are disrupted [12–14]. One of the factors that severely contribute to the aforementioned aggravation of neurons is rapid reperfusion injury following ischemic attack. Rapid reperfusion injury involves oxidative stress, leukocyte infiltration, mitochondrial mechanisms, platelet activation and aggregation, complement activation, and blood-brain barrier disruption in the brain, which result in neuronal cell death and brain dysfunction [15, 16]. Among the diverse mechanisms of action involved, oxidative damage and immune responses accompanying reperfusion are two of the most harmful results of ischemia reperfusion injury (IRI) [17]. Since inadequate oxygen is provided during ischemia, ATP generation in mitochondria is decreased; reperfusion damages the electron transport chain whereby Ca2+ overload results in mitochondrial pore opening and activation of cell death signaling cascades [18, 19]. In addition, IRI increases endothelial cell permeability and activates a variety of cytokines enhancing the inflammatory reaction, which induces postischemic brain damage [20, 21]. There have been efforts to discover new types of medications for IRI such as gold nanoparticle or herbs [22, 23]. However, there is no therapy available currently that can effectively cure the patients with cerebral IRI.

Hydrogen is a novel therapeutic substance that is mainly administered in solution in water or gas. It has been shown to be effective against cancer, lung damage, skin tumors, and liver injury by inducing anti-inflammatory, antioxidative, and antiapoptotic effects [24–27]. Hydrogen treatment is beneficial since it is easy to apply and causes no harm or pain to patients. Principally, hydrogen treatment interferes with the metabolism of reactive oxygen species (ROS), which are harmful when excessively produced. ROS are free radicals that easily react with other molecules in cells due to their instability. When ROS indiscreetly bind to molecules in the cell, it can lead to cancer, DNA or RNA damage, protein degradation, or cell death [28–32]. Molecular hydrogen is expected to bind to and stabilize unstable ROS without harming other cellular components. Since ROS production can aggravate neurodegenerative diseases, it has been worthwhile to examine the effect of hydrogen treatment in neurodegenerative diseases [33, 34]. In addition, its efficacy in several neurodegenerative diseases such as Parkinson's disease and ischemia supports the notion that molecular hydrogen is an interesting candidate as a new potential drug for IRI [35, 36]. However, research on the effect or mechanism of molecular hydrogen for IRI so far is lacking, though a few studies are revealing the molecular mechanisms of hydrogen therapy [25, 35, 37]. Therefore, in this study, we aimed to determine the effects of hydrogen treatment in mouse IRI model in terms of preventing long-term damage and recovering from the transient injury and to suggest possible molecular pathways activated by hydrogen therapy. Accordingly, hydrogen-rich water (HRW) was administered to C57BL/6 IRI mice generated by introducing bilateral common carotid artery occlusion (BCAO), which is an often-used technique to generate IRI in rodent brains [38–41]. The effects of hydrogen treatment were examined by conducting animal behavioural tests, biochemical assays, immunofluorescence analysis, and cytokine assays. Throughout those tests, we targeted the cerebral cortex, striatum, and hippocampus for injury and analyzed cytokine levels in the whole brain.

## 2. Materials and Methods

### 2.1. Animals

This study used 14-week-old (30 g) male C57BL/6 N mice (DBL, Incheon, Republic of Korea) maintained in the animal facility (*n* = 30 per group). Animals were group-housed (maximum 4 per cage). Animals were acclimated to standard laboratory conditions (12 h light/dark cycle) with food and water provided ad libitum.

### 2.2. Experimental Design

**Figure 1:**
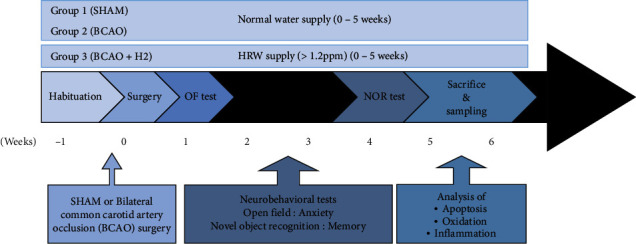
Time scheme of animal experiments. OF: open field; NOR: novel object recognition.

The experimental setup was designed to establish an acute IRI model in mice and test the efficacy of HRW treatment for 5 weeks (Figure 1). All animals underwent a week of habituation in the animal facility. Sham (*n* = 30) or BCAO surgery (*n* = 60) was performed in each group of mice, and half of the BCAO mice were randomly assigned to the HRW-treated group (*n* = 30). The HRW-treated group (H2 group) was provided with HRW immediately after the surgery; HRW was renewed every morning until the last day of experiment. After recovery from surgery, cognitive ability was tested using open field and novel object recognition tests during weeks 1 and 4. On week 5 after surgery, mice were sacrificed, and the brain samples were immediately processed for analysis. ROS assays, which are sensitive to time delays, were conducted on the same day of sacrifice. The other experimental samples were also processed immediately and analyzed as soon as possible.

### 2.3. Administration of HRW

HRW at 1.2-2.0 ppm was produced by using an HRW machine (H2B-H20, Yongkang Gomax Industry and Trade, Yongkang, China). The concentration of molecular hydrogen was measured with a dissolved hydrogen meter (ENH2000, Trustlex, Suita, Japan). Mice (*n* = 30) were assigned to each cage for group housing (maximum 4 per cage). Animals in each cage were daily provided with 300 mL HRW ad libitum from the day of surgery through the closed glass vessels coated with aluminium. HRW was freshly generated and refilled every day in the morning in between 9:00 and 10:00 AM. The volume reduction of HRW was measured every day to confirm that the mice had consumed the water.

### 2.4. BCAO Operation

Mice were anesthetized by inhalation of 5% isoflurane. Upon beginning of the surgical process, mice received 3% isoflurane through the outlet of the anesthesia machine (L-PAS-01D, LMS Korea, Pyongtaek, Korea). A midline incision was made at the ventral neck of the mice, and the common carotid arteries (CCAs) were isolated from both sides. Both arteries were occluded with bulldog clips. After 20 min of occlusion, reperfusion was initiated by removing the clips from both CCAs, ceasing isoflurane supply and sealing the incision. HRW (0.5 mL/g) was intraperitoneally injected in the H2 group, while antisepticized water was injected in the other group of mice. Once the mouse recovered normal consciousness with locomotion, it was moved back to the home cage.

### 2.5. Open Field Test

All mice were moved to a dimly lit test room 30 min prior to the test. The chamber (acryl, 50 × 50 × 30 cm^3^) was sanitized with 70% EtOH. A mouse was placed at the center of the chamber and allowed to freely explore for 10 min. The recording began 2 min after placing the animal in chamber, and its behavioural pattern was analyzed with ANY-maze software (Stoelting, Wood Dale, IL).

### 2.6. Novel Object Recognition Test

All mice were moved to the dimly lit test room 30 min prior to the test. The chamber was sanitized with 70% EtOH. Each mouse was placed in the empty chamber (acryl, 33 × 20 × 30 cm^3^) and was given 10 min for habituation. The mouse was moved to the resting cage after the habituation session. When all mice in a cage completed the session, they were moved back to the home cage. On the test day, a mouse was placed in the chamber with two identical objects located at two corners. The mouse was allowed to explore the chamber for 10 min, and then, they were moved to the resting cage. When all mice in a cage completed the task, they were moved back to the home cage. 4 hrs after the session, the mouse was placed back in the chamber for another 10 min, where one of the objects was changed to a new object. Afterwards, the mouse was returned to the home cage. The exploration behaviour of each mouse was analyzed with ANY-maze software (Stoelting).

### 2.7. Histopathological Investigation

The mouse brain sections of 6 *μ*m thickness were stained with hematoxylin and eosin and mounted on a cover glass by using a quick hardening mounting medium (03989, Sigma-Aldrich, St. Louis, MO). The slides were digitalized using Pannoramic Digital Slide Scanner (3DHistech Ltd., Budapest, Hungary).

### 2.8. Total Antioxidant Capacity Assay

Total antioxidant capacity was assessed using the OxiSelect Total Antioxidant Capacity (TAC) assay kit (STA-360, Cell Biolabs, San Diego, CA). The assay procedure was conducted according to the manufacturer's suggestion. A whole brain was thoroughly washed with 1x phosphate-buffered saline (PBS) solution. The sample was manually ground and homogenized with a polytetrafluoroethylene pestle in a 2 mL microcentrifuge tube. The sample was centrifuged at 10,000 g for 10 min at 4°C. The supernatant was collected in a fresh microcentrifuge tube. Standards of 20 *μ*L uric acid serially diluted in NaOH in the range of 0 mM to 1 mM or the samples were loaded in a 96-well plate, and 180 *μ*L 1× reaction buffer was added to each well and mixed thoroughly. The sample was initially read at 490 nm with a Synergy HTX multimode microplate reader (BioTek Instruments, Winooski, VT). Then, 50 *μ*L 1× copper ion reagent was added to each well and incubated for 5 min on an orbital shaker, followed by 50 *μ*L 1× stop solution to terminate the reaction. The plate was read again at 490 nm. Calibrations were processed in Microsoft Excel (Microsoft, Redmond, WA) based on the formula gained from the uric acid standard curve.

### 2.9. Thiobarbituric Acid Reactive Substance (TBARS) Assay

Thiobarbituric acid reactivity was assessed with the OxiSelect™ TBARS assay kit (STA-330, Cell Biolabs). Whole brain samples were centrifuged at 10,000 g for 10 min at 4°C. The supernatant was collected in a fresh 1.5 mL microcentrifuge tube, and 100× butylated hydroxytoluene (BHT) was added to each sample to achieve a 1× final concentration. Malondialdehyde (MDA) standards were serially diluted in double-distilled H_2_O in the range of 0 *μ*M to 120 *μ*M. Then, 100 *μ*L standards or samples were loaded in fresh microcentrifuge tubes, and 100 *μ*L of SDS lysis solution was added to each of them. After a 5 min incubation at room temperature (RT), 250 *μ*L TBA reagent was loaded in each tube and mixed well. All tubes were incubated at 95°C for 45 min and then incubated on ice for 5 min. Standards and samples were centrifuged at 3,000 rpm for 15 min at RT, and supernatants were collected in fresh microcentrifuge tubes. Then, 200 *μ*L of standards and samples were loaded in each well of a 96-well plate. Samples were read at 532 nm. Acquired values were processed in Microsoft Excel based on the formula gained from the MDA standard curve.

### 2.10. Western Blot

For Western blots, 40 *μ*g of protein extracted from the mouse hippocampal tissue was mixed with 5× SDS-PAGE loading dye and boiled at 95°C for 5 min in a shaking incubator (KTM-100, KBT, Seongnam, Korea). Proteins were then run at 80 V for 20 min across a 14% polyacrylamide gel, followed by 120 V for 1 hr at RT. Protein in the gel was transferred to a methanol-activated PVDF membrane at 400 mA for 40 min on ice. The membrane was blocked in 5% bovine serum albumin (BSA) (160069, MP Biomedicals, Santa Ana, CA) diluted in 1x PBS for 1 hr at RT, and then incubated with primary antibodies (Bax rabbit mAb, 14796, Cell Signaling Technology, Danvers, MA; Recombinant TNF alpha antibody, ab183218, Abcam, Cambridge, UK; *β*-tubulin mouse mAb, 86298, Cell Signaling Technology) diluted in 2.5% BSA solution with 0.2% Tween-20 at 4°C overnight. The next day, the membrane was incubated with secondary antibodies (goat anti-rabbit HRP conjugate, 170-6515, Bio-Rad, Hercules, CA; goat anti-mouse HRP conjugate, STAR207P, Bio-Rad). The conjugates were activated with enhanced chemiluminescence substrates (#1705061, Bio-Rad) and visualized under a LuminoGraph chemiluminescent imaging system (WSE-6200H, ATTO, Daejeon, Korea).

### 2.11. Immunofluorescence Microscopy

Whole mouse brains were incubated in 4% PFA for 48 hr at 4°C shortly after sacrificing. Brain specimens were washed several times in 1× PBS and cryoprotected in 50% sucrose buffer for 72 hr. Frozen brains were sectioned at 8 *μ*m and blocked in 1% BSA diluted in PBS. The sections were incubated with TNF*α* primary antibody (ab183218, Abcam) diluted in 1% BSA solution for 1 hr at RT, and then incubated with secondary antibody conjugated with Alexa Fluor 568 goat anti-rabbit IgG (F0257, Sigma-Aldrich, St. Louis, MO) for 1 hr at RT. Mounting medium with DAPI (H-1200 Vectashield, Vector Labs, Burlingame, CA) was spread over the section, and the specimen was mounted with a cover glass. Immunolabeled proteins were detected by fluorescence microscopy (DMI8+DFC450, Leica Biosystems, Wetzlar, Germany).

### 2.12. Cytometric Bead Array (CBA) Assay

A cytometric bead array (CBA) kit for mice was purchased (51-9006250, BD Biosciences, Franklin Lakes, NJ). Mouse whole brain tissues were mechanically dissociated and filtered through a 40 *μ*m cell strainer (CLS431750, Corning Inc., Corning, NY). The collected samples were centrifuged at 300 g for 5 min. Pellets were fixed with 2% PFA for 30 min, incubated with 0.3% Triton X100 for 20 min at RT, and blocked with 2% BSA for 30 min on ice. At every step, cells were washed with 1× PBS and centrifuged at 300 g for 5 min to collect cell pellets. The following procedures were conducted as per the manufacturer's instructions. Briefly, lyophilized mouse standards were serially diluted from 20 to 5000 pg/mL, and the standards or samples were incubated with the capture beads and detection reagents. These were incubated for 2 hr at RT in the dark and then diluted with wash buffer and centrifuged. The supernatant was decanted and 300 *μ*L wash buffer was loaded to each standard or sample. The final products were read with a flow cytometer (Cytoflex 2L6C, Beckman Coulter, Brea, CA).

### 2.13. Statistical Analysis

Data was shown as mean ± standard error of the mean in all figures. One-way analysis of variance (ANOVA) was used for comparisons of three groups, and Tukey's multiple comparison test was used as a post hoc test, except for ROS analysis which used a Newmann-Keuls post hoc test. Prism5 software (GraphPad Software, San Diego, CA) was utilized for analysis. A minimum *p* value of <0.05 was considered statistically significant.

## 3. Results

### 3.1. Pyknotic Cells Shown by Hematoxylin and Eosin Staining in the Corpus Callosum, Striatum, and Cortex in Each Group of Mouse Brain

**Figure 2:**
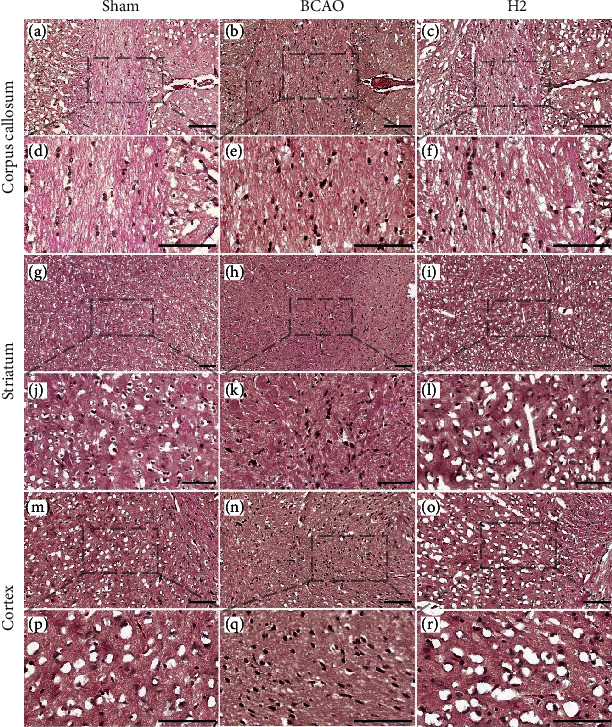
The representative images with HE stained 6 *μ*m thick coronal brain sections obtained from each group for comparison in 2D imaging. Scale bar = 100 *μ*m. The dashed line and box show the magnified regions. (a–c) Corpus callosum, 40× magnified; (d–f) corpus callosum, 100× magnified; (g–i) striatum, 20× magnified; (j–l) striatum, 63× magnified; (m–o) cortex, 40 × magnified; and (p–r) cortex, 100× magnified.

To detect cellular changes in the mouse brain after IRI or hydrogen treatment, hematoxylin and eosin (HE) staining was conducted after sacrifice of animals (Figure 2). The corpus callosum, striatum, and cortex were investigated as a target region of IRI attack. A larger number of darker pyknotic cells were detected in the brain of BCAO model mouse in all regions compared to that of the sham or H2 group, whereas it was most strongly shown in the cortex. The pyknosis seemed to be attenuated in the brain of H2 treated mouse in all regions.

### 3.2. Molecular Hydrogen Restores Cognitive Functions of Anxiety and Memory in Mice

**Figure 3:**
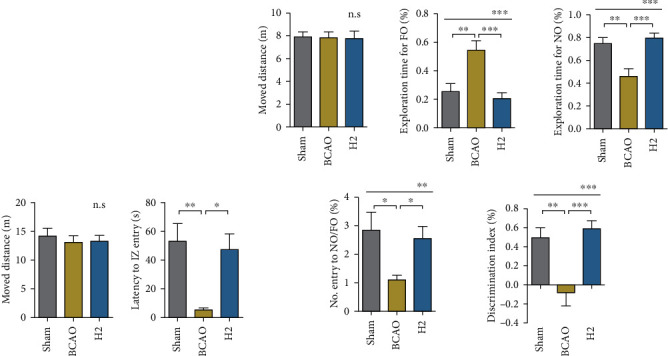
Results of open field and novel object recognition tests. (a) Mice were placed in an open field chamber, and the patterns of movement were analyzed (*n* = 13 per group). IZ: inner zone. (b) Mice were placed in a chamber where a familiar and a novel object were each located at different corners. No. Entry to NO/FO (%): the number of entries to the novel object area compared to the entries to the familiar object area; discrimination index (%): the level of recognizing, remembering, and discriminating the novel object from the familiar object. (*n* = 18, 18, and 16 for sham, BCAO, and H2, respectively). NO: novel object; FO: familiar object; BCAO: bilateral common carotid artery occlusion. ^∗∗∗^*p* < 0.0001, ^∗∗^*p* < 0.01, and ^∗^*p* < 0.05; n.s: not significant.

To examine the impairment of anxiety in our models, an open field test was conducted, focusing on how fast the mice crossed a line to enter the inner zone (IZ) without maintaining a peripheral preference. Locomotor ability did not affect the test result, as shown in Figure 3(a) (*p* = 0.7629). The latency time to enter the IZ was measured to assess whether mice experienced the normal level of anxiety. A significant difference in the latency time of the first entry to the IZ was observed between groups (*p* = 0.0032; ANOVA). In the post hoc analysis, a significant difference in the latency time to enter the IZ was observed between the sham and BCAO groups (*p* < 0.01). Mice in the H2 group showed recovered anxiety levels that were significantly different from those of the BCAO group (*p* < 0.05).

Next, the novel object recognition test was conducted to examine the learning and memory function in mice (Figure 3(b)). Locomotor ability did not affect the test results, as shown in the graph of moved distance (*p* = 0.9705). The exploratory time for the familiar object differed between groups (*p* = 0.0003, ANOVA), with the significant differences in post hoc analysis between the sham and BCAO groups (*p* < 0.01) and between the H2 and BCAO groups (*p* < 0.001). The exploratory time for the novel object also differed between groups (*p* = 0.0003), with the significant differences in post hoc analysis between the sham and BCAO groups (*p* < 0.01) and between the H2 and BCAO groups (*p* < 0.001). There was no significant difference between the sham and H2 groups. Furthermore, the number of entries to the novel object area in comparison to that of the familiar object area was calibrated in each group. The mean values significantly differed between groups (*p* = 0.0096, ANOVA). In the post hoc analysis, the significant differences were identified between the sham and BCAO groups (*p* < 0.05) and between the H2 and BCAO groups (*p* < 0.05), whereas there was no difference between the sham and H2 groups. The discrimination index was calculated by the formulae as follows: ((Exploration time for novel object − Exploration time for familiar object)/(Exploration time for novel object + Exploration time for familiar object)). We found that the mean discrimination index significantly differed between groups (*p* = 0.0003, ANOVA), with the BCAO group having the lowest value. In the post hoc analysis, significant mean differences between the sham and BCAO groups (*p* < 0.01) and between the H2 and BCAO groups (*p* < 0.001) were identified.

### 3.3. Molecular Hydrogen Reduces ROS Levels in Mice

**Figure 4:**
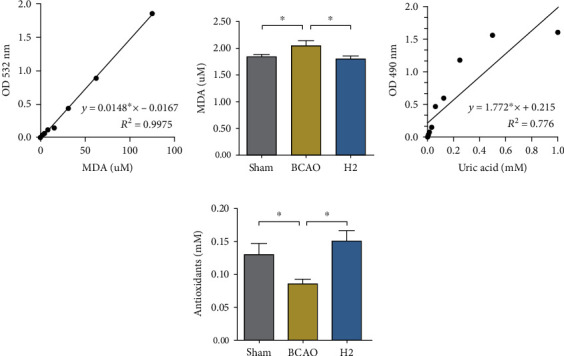
Malondialdehyde and total antioxidant levels in whole mouse brains. (a, c) Standard reference samples (MDA or uric acid) were serially diluted in certain ranges. Standard curves are presented with equations and *R*^2^ values. (b, d) Mouse whole brain samples incubated with the proper reagents were read at 532 nm (MDA) or 490 nm (antioxidants) for the calibration of quantity, and values were calculated according to the equations of the standard curves (*n* = 7 in sham and BCAO groups, *n* = 8 in H2 group). MDA: malondialdehyde; BCAO: bilateral common carotid artery occlusion; TBARS: thiobarbituric acid reactive substance; TAC: total antioxidant capacity; ^∗^*p* < 0.05.

To examine the antioxidative role of HRW in IRI, ROS concentration of the whole mouse brain was analyzed using MDA and TBARS assay kits (Figure 4). The TBARS assay was performed to quantify MDA level, a measure of lipid peroxidation. The TAC assay was also performed to evaluate the concentration of the total antioxidants that scavenge free radicals. The MDA concentration was significantly higher in the BCAO group compared to the other groups (*p* < 0.0152, ANOVA). In the post hoc analysis, significant differences were observed between both the sham and BCAO groups and between the H2 and BCAO groups (*p* < 0.05), while no significant difference was observed between the sham and H2 groups (Figure 4(b)). Contrastingly, the total antioxidant capacity was significantly lower in the BCAO group compared to the other groups (*p* < 0.0408, ANOVA). In the post hoc analysis, significant differences were observed between both the sham and BCAO groups and between the H2 and BCAO groups (*p* < 0.05), whereas no significant difference was observed between the sham and H2 groups (Figure 4(d)).

### 3.4. Molecular Hydrogen Attenuates Apoptosis in the Striatum and Cerebral Cortex

**Figure 5:**
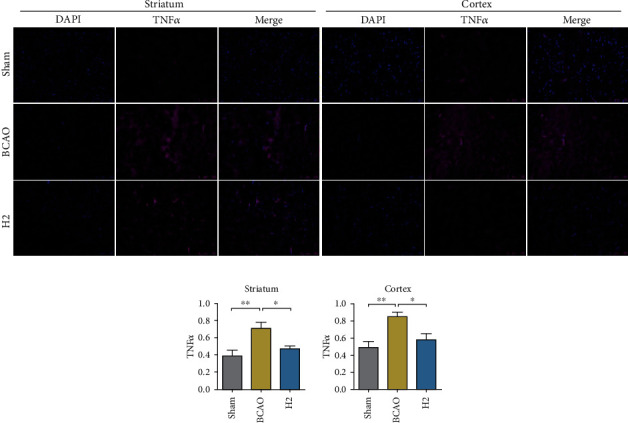
Immunofluorescence data of TNF*α*- and DAPI-stained mouse brain slices and bar graphs displaying measurements. (a) Mouse brain slices were stained with TNF*α* (magenta) and DAPI (blue), and regions of the striatum and cerebral cortex were visualized under a microscope (20× magnification). (b) Bar graphs displaying immunofluorescence measurements. The intensity of TNF*α* was calibrated in proportion to DAPI in each sample (*n* = 8, 7, and 9 for sham, BCAO, and H2 groups, respectively). BCAO: bilateral common carotid artery occlusion; ^∗∗^*p* < 0.01, ^∗^*p* < 0.05.

To analyze a cell death marker in the cerebral cortex and striatum, the mouse brain slices were fluorescently stained with TNF*α* antibody. The striatum and cerebral cortex regions were individually visualized under a microscope (×20) (Figure 5(a)). During quantification, a significant mean difference in TNF*α* concentration was observed between groups (*p* = 0.0026 for the striatum, *p* = 0.0084 for the cerebral cortex; ANOVA). In the post hoc analysis, the mean TNF*α* concentration was significantly higher in the BCAO group than in the sham group in both regions (*p* < 0.01, respectively). The H2 group showed a significantly lower TNF*α* concentration in both regions compared to the BCAO group (*p* < 0.05, respectively) (Figure 5(b)).

### 3.5. Molecular Hydrogen Attenuates Extrinsic and Intrinsic Apoptosis in the Hippocampus

**Figure 6:**
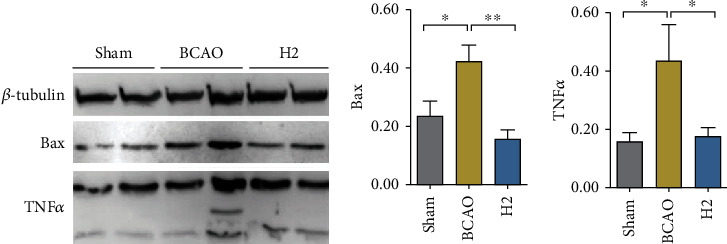
Immunoblot analysis of Bax and TNF*α* in the mouse brain hippocampus and bar graphs displaying measurements. (a) *β*-Tubulin, Bax, and TNF*α* in the mouse hippocampus were transferred to a membrane and visualized. Two bands per group are presented. (b) Bar graphs displaying immunoblot measurements of Bax quantification and TNF*α*. Both were normalized by *β*-tubulin measurements (*n* = 8 for each group); ^∗∗^*p* < 0.01, ^∗^*p* < 0.05.

To further investigate protein concentration in the hippocampus, Western blot analysis was conducted (Figure 6). A significant difference in Bax concentration was observed between groups (*p* = 0.0024, ANOVA). The mean Bax value in the BCAO group was significantly higher than in the sham or H2 groups in post hoc analysis (*p* < 0.05, *p* < 0.01, respectively). A significant mean difference in the TNF*α* concentration was also observed between groups (*p* = 0.0151, ANOVA). The mean TNF*α* concentration in the BCAO group was significantly higher than that in the sham and H2 groups in post hoc analysis (*p* < 0.05, respectively).

### 3.6. Potential Molecular Mechanisms of Inflammation by Hydrogen Treatment via Cytokine Expression

**Figure 7:**
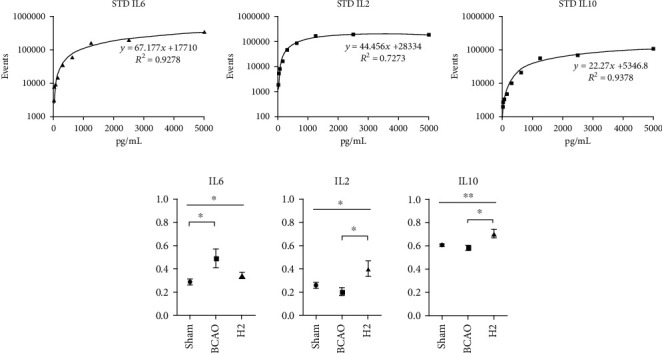
Standard curves fit to calibration data from serially diluted standard references and comparisons of IL-6, IL-2, and IL-10 between groups for whole mouse brain. (a) Standard curves of diluted IL-6, IL-2, and IL-10. (b) Comparisons of cytokine concentrations between groups (*n* = 12, 10, and 12 for sham, BCAO, and H2 groups, respectively). BCAO: bilateral common carotid artery occlusion; ^∗∗^*p* < 0.01, ^∗^*p* < 0.05.

To investigate the potential molecular mechanisms involved with the inflammatory pathways in the central nervous system targeted by hydrogen treatment, several cytokines were selected and quantified in the mouse brain (Figure 7). Each standard reference cytokine sample was serially diluted between 0 and 5000 pg/mL. Logarithmic graphs of concentrations were plotted and are shown in Figure 6(a). Based on the equation acquired from the slope of the trendline, cytokine concentrations were calculated, and they are presented in Figure 7(b). There were significant differences in IL-6, IL-2, and IL-10 levels between the sham, BCAO, and H2 groups (*p* = 0.0286, 0.0156, and 0.0097, respectively; ANOVA). A significant difference was observed in IL-6 between the sham and BCAO groups in post hoc analysis (*p* < 0.05), and significant differences in IL-2 and IL-10 were observed between the BCAO and H2 groups (*p* < 0.05, respectively).

## 4. Discussion

Molecular hydrogen is a novel therapeutic substance that is easy to administer and is powerful in its chemical reactivity. While previous studies have predominantly used hydrogen sulfide to evaluate diseases including ischemia, investigation of the role of molecular hydrogen in cerebral IRI has not been sufficiently explored [42–44]. To broaden its application and test the efficacy of hydrogen therapy, this study is aimed at verifying the effect of orally ingested HRW in IRI mice and identifying the potential molecular mechanisms involved with inflammatory pathways in the cerebral cortex using behavioural tests, biochemical assays, and immunofluorescence microscopy techniques. The results showed the potential of hydrogen therapy as a treatment for IRI, and we further identified the possible mechanisms of action.

In the present study, anxiety behaviours and memory were assessed to evaluate the effect of molecular hydrogen against IRI, since memory loss and abnormal anxiety behaviours are the prominent symptoms in IRI [45, 46]. To test these cognitive functions in mice, two different neurobehavioural tests were introduced. For analysis of anxiety, the open field test was conducted after a week of rest from surgery. Previous literature has shown that ischemic rodents spend more time in an open area compared to the sham-operated animals due to the decreased anxiety [47]. Another study showed that ischemic rodents moved irregularly and hyperactively across zones, whereas control mice primarily stayed in the peripheral zones [48]. Both the aforementioned studies demonstrate that an abnormal anxiety pattern is observed in the ischemic mouse behaviour. In our research, the distinctive anxiety level with hyperactivity and loss of the peripheral preference in the BCAO group was observed; HRW therapy restored anxiety back to the normal level in this mouse model. Secondly, the novel object recognition test was conducted to assess memory restoration and hippocampal function. The results showed obvious differences between groups in terms of the ability to discriminate the objects. Regarding the entries to the novel object or familiar object zones, the sham group tended to preferably explore the novel object, whereas this behaviour was significantly impaired in the BCAO group. However, this impairment was attenuated in HRW-treated mice, and no significant difference was found when compared to the sham mice. Both behavioural test results support that BCAO surgery induced abnormal anxiety and memory deficits in mice as one of the prominent symptoms shown in IRI and that HRW attenuates these cognitive impairments. Besides, the cell deaths detected in the HE-stained brain slice of IRI model mouse, distinct from those of the sham and H2-treated mouse brain, further support the model establishment in our study; it also supports the plausibility of the protective effect of molecular hydrogen in cerebral IRI.

Since IRI is accompanied with an increase of ROS concentration in the brain, which often leads to other complex neurodegenerative diseases, the present study evaluated the ROS concentration in the whole mouse brain and demonstrated it with the total antioxidants and lipid peroxidation indices. The results demonstrated that the lipid peroxidation was increased, and the antioxidants level was decreased in IRI model mice, whereas the ability of HRW to alleviate ROS activity was clearly observed with the recovered antioxidant and attenuated lipid peroxidation levels in HRW-treated mice. These results identified the anti-oxidative effect of molecular hydrogen in IRI model mice, which is in agreement with the previous studies that showed the role of molecular hydrogen in scavenging free radicals or interfering with ROS generation [27, 49–51]. In addition, our findings found the connection between the effects of ROS removal with antiapoptosis, by selecting lipid peroxidation level as an index that causes neuronal cell death [52]. This antioxidation effect of molecular hydrogen is anticipated to contribute to neuroprotection in IRI.

To further examine the antiapoptotic effect of HRW in IRI model mice, we assessed the TNF*α* concentration in a mouse brain by immunofluorescence analysis, which shows the extrinsic apoptosis level in tissues [53]. Our finding supports that HRW greatly reduces TNF*α* levels, contrary to the abundant TNF*α* observed in the cerebral cortex and striatum of IRI model mice, which is in accordance with the recovery pattern after IRI shown in the earlier studies [54, 55]. We further quantified the antiapoptotic role of molecular hydrogen in the hippocampus, which is a core region for learning and memory, with the Bax and TNF*α* concentrations, each indicating the intrinsic and extrinsic cell death stimulation index [56, 57]. According to the immunoblot analysis, these apoptotic signaling molecules were highly expressed in the hippocampi of IRI model mice, whereas they were reduced in the HRW-treated mouse brains. Given these results, the antiapoptotic function of molecular hydrogen is supposedly not restricted to the extrinsic apoptotic pathway, but further involves the inhibition mechanism against the intrinsic apoptotic pathway in the hippocampus, which is closely related to ROS production in mitochondria [58, 59]. It is presumed that molecular hydrogen ultimately prevents cell death in the striatum, cerebral cortex, and hippocampal tissues after IRI, in a complex manner integrating ROS regulation and antiapoptotic action.

Finally, a cytokine assay was conducted to monitor the effect of HRW on immune pathways in the mouse brain. According to the previous research, molecular hydrogen is known to reduce inflammatory cytokines, including IL-6 and TNF*α*, which are the critical proinflammatory cytokines [51, 60]. In addition, our data suggests that IL-6 is acutely overexpressed after IRI introduction, and HRW therapy induces the overexpression of IL-2 and IL-10 after IRI. Given the detailed mechanisms of action of these cytokines, it seems that molecular hydrogen affects cells in an organized manner, orchestrating activation of IL-2 and IL-10 to regulate inflammation via regulatory T cells (Tregs) that are involved in regulating autoimmunity and suppressing immune activation [61]. IL-2 interferes with IL-6-dependent signaling events to control inflammation by inhibiting Th17 differentiation [62–64]. IL-10 restricts inflammation-mediated damage by directly blocking the proinflammatory Th17 proliferation [62–66]. Likewise, our findings support the notion that hydrogen therapy possibly works by promoting Tregs to reduce the activation of proinflammatory cytokines.

Our study highlighted the potential of hydrogen molecule to serve as a novel medication to attenuate the transient injury and prevent a long-term damage after cerebral IRI, which is distinguishable from previous studies that only focused on the acute injuries [67–69]. Furthermore, we suggested the novel inflammatory pathway that could possibly be involved with the mechanism of action of molecular hydrogen treatment. However, a small number of samples limited the statistical power in this research, and only a few number of protein candidates that were analyzed in immunoblots and immunofluorescence generate a missing gap in the signaling dynamics; this limits our understanding of the detailed mechanisms of molecular hydrogen therapy. To make up for these limitations, a bigger number of protein candidates must be analyzed to assess its participation in the H2 therapy mechanism in future studies. Besides, more samples should be assigned to each group to result in more credible conclusions, and a quantitative data for histopathological analysis should be included in future studies. This will greatly help improve our understanding about the molecular hydrogen therapy, and it will also help us to understand the therapeutic progress of the disease model to ultimately contribute to accurate biomarker selection in the future.

## 5. Conclusions

This study is aimed at verifying the effect of molecular hydrogen on ischemic reperfusion injury in mice and identifying the possible mechanism of action. There have been several findings that confirm that hydrogen sulfide attenuates apoptosis and neuronal injury in the hippocampus [49, 66] and that molecular hydrogen attenuates autophagic cell death via the FoxO1 pathway in mice with vascular dementia [9]. While those findings provide foundations for understanding the antioxidational or antiautophagic effect of hydrogen therapy, the effect of molecular hydrogen on inflammation and other detailed mechanisms in cells are still unclear, especially when it comes to neurodegenerative diseases. In this regard, our research not only verified the functions of molecular hydrogen in the ischemic reperfusion injury, one of the prominent neurodegenerative diseases, but also proposed its possible mechanism of action by suggesting the specific T cell differentiation as the candidate.

In summary, hydrogen therapy improves cognitive ability and induces antioxidative and antiapoptotic effects in the ischemic reperfusion injury model mouse. Moreover, molecular hydrogen potentially induces anti-inflammatory effects through Tregs. These findings will provide direction for further investigations.

## Data Availability

The data that supports the findings of this study will be available from the corresponding author on reasonable request (Jong-Il Choi; thlthd@korea.ac.kr).
